# A Comprehensive Review of Novel Advances in Type 1 Diabetes Mellitus

**DOI:** 10.1111/1753-0407.70120

**Published:** 2025-08-06

**Authors:** Yazdan Ebrahimpour, Sahbasadat Khatami, Mahsa Saffar, Alireza Fereidouni, Zahra Biniaz, Nafiseh Erfanian, Mohammad Fereidouni

**Affiliations:** ^1^ Student Research Committee Birjand University of Medical Sciences Birjand Iran; ^2^ Cellular and Molecular Research Center Birjand University of Medical Sciences Birjand Iran

**Keywords:** gene therapy, immunotherapy, Type 1 diabetes mellitus

## Abstract

Type 1 diabetes mellitus (T1DM) is a chronic autoimmune disorder where the immune system targets and destroys insulin‐producing β‐cells in the pancreas. It generally emerges during childhood or adolescence, but individuals of any age can be affected. In contrast to Type 2 diabetes mellitus (T2DM) which is often associated with lifestyle factors, T1DM cannot be prevented and necessitates lifelong management. Currently, there is no definitive cure for T1DM, and patients rely on continuous insulin injections for their entire lives. Ongoing developments in insulin treatment, such as insulin pumps, continuous glucose monitoring, and hybrid closed‐loop systems, offer promising alternatives. Despite advancements in intensive glycemic control that have reduced the occurrence of microvascular and macrovascular complications, a significant number of T1DM patients still these issues. Extensive research efforts are crucial to achieve early detection, prevent the loss of β‐cells, and devise improved treatment strategies to enhance the quality of life and prognosis for those affected. This review explores the most noteworthy and recent advancements in the field of T1DM.

## Graphical abstract

Novel approaches in the way of T1DM treatment.

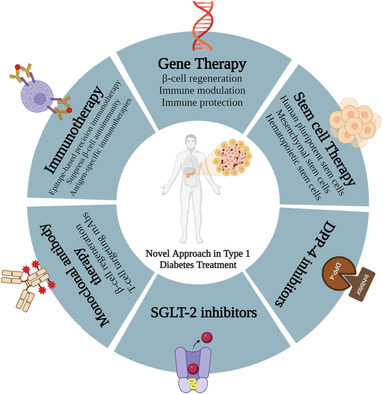

**Summary**

- The treatment landscape for T1DM is experiencing significant change with the development of innovative therapies like SGLT‐2 and DPP‐4 inhibitors, gene therapy, stem cell therapy, and immunotherapy.
- These advancements offer potential for better glycemic control, β‐cell regeneration, and immune modulation, positioning T1DM care at the forefront of precision treatment.
- Despite challenges, ongoing research and collaboration hold promise for improved outcomes, offering hope for a brighter future for those managing this autoimmune condition.

## 1 Introduction

Type 1 diabetes mellitus (T1DM), also referred to as autoimmune diabetes, is a persistent condition marked by a deficiency of insulin due to the loss of pancreatic β‐cells, resulting in hyperglycemia. Although symptoms typically manifest during childhood or adolescence, they can also emerge later in life. While the exact cause of T1DM remains not entirely clear, the disease's pathogenesis is believed to involve the T cell‐mediated destruction of β‐cells [1]. Islet‐targeting autoantibodies directed against insulin, 65 kDa glutamic acid decarboxylase (GAD65) [2], insulinoma‐associated protein 2 (INSM2) [3], and zinc transporter 8 (ZnT8)—proteins associated with secretory granules in β‐cells—serve as biomarkers for T1DM‐related autoimmunity. These autoantibodies are detectable months to years before the onset of symptoms, offering a means to identify and study individuals at risk of developing T1DM [4].

While commonly diagnosed during childhood or adolescence, this condition can affect individuals of any age, distinguishing it from Type 2 diabetes (T2DM), which often stems from lifestyle factors [5]. Symptoms such as increased thirst, frequent urination, extreme hunger, unexplained weight loss, fatigue, and blurred vision underscore the critical need for meticulous blood sugar control. The cornerstone of T1DM management is insulin therapy, administered through multiple daily injections or insulin pumps [6]. On the other hand, lifestyle adjustments, encompassing a well‐balanced diet and regular exercise, play pivotal roles in effective T1DM management [7].

Balancing blood sugar levels not only prevents short‐term complications like hypoglycemia but also mitigates the risk of long‐term complications, including heart disease [8] and nerve damage [9]. However, despite notable progress in treatment options, individuals with T1DM still confront challenges. The psychological impact of constant monitoring and adherence to treatment regimens necessitates a comprehensive approach, emphasizing the significance of psychosocial support and education [10]. As such, ongoing research into new treatment methods is essential to address these challenges comprehensively. This paper aims to explore the critical importance of ongoing research in unveiling innovative approaches to treating T1DM. By actively addressing existing limitations, these advancements have the potential not only to revolutionize diabetes management but also to significantly enhance patient care and contribute valuable insights to the scientific understanding of this complex condition.

## 2 Understanding the Multifaceted Role in T1DM Development

While the exact etiology of T1DM remains elusive, it is widely accepted that multiple factors contribute to its onset and progression (Table 1).

**TABLE 1:** The genetic factors that play a role in T1DM.

Genetic factor	Genes/alleles	Role in T1DM	References
HLA Class I genetic factors	HLA Class I genes (A, B, C)	Bind to CD8+ T lymphocytes, contributing to T1DM risk	[15]
	HLA‐A24	Associated with limited or absent remaining β‐cell function	
	Additional alleles (e.g., A*02:01, B*18:01, C*05:01, A*11:01, A*32:01, A*66:01, B*07:02, B*44:03, B*35:02, C*16:01, C*04:01)	Predisposing or protective factors for T1DM	
HLA Class II genetic factors	HLA Class II genes (DR, DQ)	Major genetic factor associated with T1DM	
	HLA‐DRB1*03:01 (DR3) and HLA‐DRB1*04:01/02/04/05/08 (DR4)	Highest risk, often seen in haplotypic association	
	HLA‐DRB1*04 (DR4*) in haplotypic association with *DQA1*03‐DQB1*03:01 (DQ7)	Not linked to a significant risk for T1DM	
	HLA‐DR4/DR3 heterozygotes	Extremely high vulnerability	
	HLA‐DPB1 alleles	Susceptibility and protective factors in various ethnic groups	
Non‐HLA genetic factors	INS gene (on chromosome 11p15.5)	Contributes approximately 10% to T1DM hereditary vulnerability	[17]
	PTPN22 gene (on chromosome 1p13)	Codes for LYP, associated with T1D susceptibility	[19]
	CTLA‐4 gene (on chromosome 2q33)	Polymorphisms linked to T1DM, such as +49A/G	[18]

Abbreviations: CTLA‐4: cytotoxic T‐lymphocyte–associated antigen 4; HLA: human leukocyte antigens; INS: insulin gene; LYP: lymphoid phosphatase; PTPN22: protein tyrosine phosphatase nonreceptor Type 22; T1DM: Type 1 diabetes mellitus.

### 2.1 Role of Gut Microbiota in T1DM

The human gut microbiota has emerged as a crucial factor in the development of various diseases, including T1DM. This complex microbial ecosystem, composed of trillions of microorganisms, plays an essential role in regulating the immune system [11]. Dysbiosis, an imbalance in the gut microbiota, can compromise the intestinal barrier by disrupting tight junctions, resulting in increased intestinal permeability, often referred to as “leaky gut.” This condition allows microbial antigens, such as lipopolysaccharides (LPS), to translocate into the bloodstream, triggering systemic inflammation [12]. Elevated levels of LPS have been linked to insulin resistance and beta‐cell dysfunction, both of which contribute to the pathogenesis of T1DM [13].

Toll‐like receptors (TLRs), key pattern recognition receptors, recognize microbial components and initiate innate immune responses. MyD88, an essential adaptor protein in TLR signaling, plays a central role in this pathway. Studies have demonstrated that NOD mice deficient in MyD88 are protected from developing T1DM, suggesting that interactions between the gut microbiota and TLRs influence autoimmune responses [14]. Microbial antigens are processed by antigen‐presenting cells, such as dendritic cells and macrophages, which subsequently activate autoreactive T cells. This interaction promotes the differentiation of naïve T cells into pro‐inflammatory subsets, particularly, Th1 and Th17 cells, both of which are involved in beta‐cell destruction [15]. Additionally, gut microbiota modulates the development and function of regulatory T cells (Tregs), which are critical for maintaining immune tolerance [16]. A reduction in Treg populations or dysfunction of Tregs exacerbates autoimmune responses, accelerating beta‐cell destruction [17].

Emerging evidence highlights the involvement of specific gut bacteria in T1DM. Certain commensal bacteria express glutamic acid decarboxylase (GAD) [18] and produce gamma‐aminobutyric acid (GABA), both of which influence intestinal barrier integrity and immune modulation [19]. Moreover, short‐chain fatty acids (SCFAs), particularly butyrate, produced by the gut microbiota during fiber fermentation, are essential for maintaining gut integrity and regulating immune responses [20]. Butyrate enhances Treg function through histone modification, thereby promoting immune tolerance [21]. A decrease in SCFA‐producing bacteria has been observed in individuals with T1DM, further emphasizing the link between microbial metabolites and beta‐cell autoimmunity [22].

A notable feature of gut dysbiosis in T1DM is a reduced Firmicutes/Bacteroidetes (F/B) ratio, a trend observed across various populations, including those in China [23] and Turkey [24]. Metagenomic studies have also revealed a decline in butyrate‐producing bacteria, mucin‐degrading species, and Bifidobacterium in T1DM patients [25, 26]. Significantly, early‐life interventions such as *Bifidobacterium infantis* supplementation and probiotic administration have shown promising effects in reducing hemoglobin A1c (HbA1c) levels and decreasing insulin requirements [27].

### 2.2 Role of Genetic Factors in T1DM

Genetic predisposition stands as a significant contributor to T1DM. Numerous investigations have indicated that as much as 50% of T1DM susceptibility can be attributed to inherited factors [11]. While the general populace faces a lifetime risk of around 0.4% for T1DM, individuals with familial ties to T1DM patients encounter an elevated likelihood of developing the disorder. Over 60 genes have been identified to potentially contribute to the onset of T1DM, which can be classified into three main categories, including HLA genetic factors (Classes I and II) and non‐HLA genetic factors [12].

#### 2.2.1 HLA Genetic Factors

The HLA genes are the major genetic factor associated with the disease. HLA complex plays a critical role in the pathogenesis of T1DM. The association between T1DM and HLA was first reported in 1973, following the observation of increased frequency of HLA antigens in T1DM patients, compared with those in controls [13].

– *HLA Class II*: The HLA class II genes at 6p21 map to the main T1DM susceptibility locus, which is responsible for up to 30%–50% of the hereditary risk of the disease. It is generally established that alleles at HLA loci, particularly, the HLA class II genes DR and DQ, are associated with T1DM [14].
– *HLA Class I*: HLA class I genes, such as HLA‐A, HLA‐B, and HLA‐C, bind to CD8+ T lymphocytes and provide them with peptide antigens. They have a dual role in thymus‐based T‐cell repertoire formation and antigen‐specific response initiation, offering a strong immunological explanation for the genetic correlation with T1DM [15].

#### 2.2.2 Non‐HLA Genetic Factors

Comparatively to HLA, the combined effects of other non‐HLA T1DM loci on illness risk are less significant [16]. These include the insulin gene (INS)3 on chromosome 11p15 [17], the polymorphic, cytotoxic T‐lymphocyte‐associated protein 4 (CTLA4) gene on chromosome 2q33 [18], the protein tyrosine phosphatase nonreceptor Type 22 (PTPN22) gene on chromosome 1p13 [19], the Interleukin 2 receptor, alpha (IL2RA) [20], interferon induced with helicase C domain 1 (IFIH1) genes [21] and other recently found loci from genome‐wide association (GWA) research.

## 3 Novel Approaches for T1DM Treatment

The landscape of T1DM management is undergoing a transformative evolution, marked by the exploration of innovative therapies and novel approaches. As researchers delve into cutting‐edge developments, a spectrum of promising interventions emerges, ranging from advanced technological solutions to revolutionary biological and genetic treatments.

### 3.1 Gene Therapy

Gene therapy approaches for T1DM can be broadly categorized into three main strategies: β‐cell regeneration, immune modulation, and immune protection.

#### 3.1.1 β‐Cell Regeneration

The primary objective of gene therapy in T1DM is to restore β‐cells that have been damaged or eliminated by the disease. To this end, various strategies have been investigated, including the use of transcription factors to convert other cell types into β‐cells. Research has demonstrated that compulsory expression of pancreatic duodenal homeobox 1 (PDX1) and neurogenin 3 (NEUROG3) can transform liver cells into functional insulin‐secreting β‐like cells [22].

#### 3.1.2 Immune Modulation

T1DM is an autoimmune disease, and gene therapy can be employed to regulate the immune system and prevent the destruction of β‐cells. One approach involves the use of gene‐editing tools, such as CRISPR‐Cas9, to selectively modify immune cells. CRISPR has been successfully used to disrupt genes associated with autoimmunity, thereby preventing the destruction of β‐cells in animal models [23].

#### 3.1.3 Immune Protection

Another strategy in gene therapy for T1DM is protecting newly generated or transplanted β‐cells from immune attack. A promising strategy is the genetic engineering of β‐cells to evade immune detection. Studies have explored the use of chimeric antigen receptor regulatory T cells (CAR‐Tregs) to selectively protect stem cell‐derived β‐cells from immune destruction. Engineering β‐cells to express truncated epidermal growth factor receptor (EGFRt) allows CAR‐Tregs to target and shield them from autoreactive immune responses [24]. Additionally, manipulating molecular pathways involved in antigen presentation has been proposed to “cloak” β‐cells from T‐cell recognition, potentially improving long‐term graft survival [25]. Another approach involves biomaterial‐based immune protection. Advances in microencapsulation techniques now allow the development of biocompatible materials that create an immune barrier while preserving nutrient exchange, improving graft survival [26]. Furthermore, nanoscale drug delivery systems can locally release immunomodulatory agents to suppress autoreactive T‐cell activity, reducing the need for systemic immunosuppression [27].

Despite these advances, gene therapy still faces challenges, including long‐term gene expression stability, immune responses against viral vectors, and cost‐effectiveness. Human immune reactions to viral vectors may compromise therapeutic efficacy, necessitating safer and more precise delivery methods [28, 29]. Recent breakthroughs, such as CRISPR‐based gene editing and ViaCyte's VCTX210—a gene‐edited, stem cell‐derived therapy—have shown efficacy in restoring insulin production without immunosuppression in early stage T1DM patients. However, their effectiveness in long‐term disease management remains uncertain due to patient variability in immune response and disease progression [30].

### 3.2 Stem Cell Therapy

In T1DM, autoimmune destruction of islet β‐cells is the causative factor. Also, Islet β‐cell transplantation has been considered a curative treatment in T1DM, but limited donor supply and immune rejection of transplanted cells restrict the use of this strategy [31]. Stem cell therapy offers a potential solution by aiming to regenerate or replace the damaged β‐cells and reconstitute immunotolerance and preservation of islet β‐cell function [32]. Until now, different types of stem cells and varied strategies have been used for differentiation and engraftment of stem cells. The most common types of stem cells used for restoring β‐cell functions are as follows:

### 3.3 Human Pluripotent Stem Cells (hPSCs)

hPSCs can replicate unlimitedly and differentiate from any adult cell type. Several studies confirmed the successful differentiation of hPSCs to mature pancreatic endocrine cells and insulin‐producing cells [33, 34]. Intensive studies have been conducted for the generation of IPCs (induced pluripotent stem cells) or islet organoids in vitro since hPSCs have been anticipated for application in regenerative medicine. The sources for this regeneration mainly include hPSCs, human embryonic stem cells (hESCs), human induced pluripotent stem cells (hiPSCs), adult stem cells, and differentiated cells from mature tissues. However, transplantation of IPCs derived from hESCs has two major side effects, including teratogenicity and immunogenicity. To overcome these limitations, some researchers used immunoisolation devices and/or immunosuppressive agents, but the results of clinical trials showed minimal cell survival and a lack of a significant clinical benefit [32, 35].

#### 3.3.1 Mesenchymal Stem Cells (MSCs)

MSC cells exist in various tissues and play a crucial role in tissue repair following injuries. They offer several advantages, including ease of expansion, lack of HLA Class II antigens, and immunomodulatory functions [36]. While some studies have demonstrated the ability of MSC cells to promote islet graft vascularization and support pancreatic islet repair [37], others have reported challenges in their engraftment [32]. Transplanting MSCs alongside autologous islet cells has been associated with observed improvements in quality of life and graft survival. A recent systematic review and meta‐analysis indicated that MSC infusion led to improved HbA1c levels, although no significant differences were observed in fasting glucose or C‐peptide levels [38]. Despite the potential benefits of MSC cells, numerous uncertainties persist, highlighting the need for further high‐quality, large‐scale studies on their use in treating T1DM [32].

#### 3.3.2 Hematopoietic Stem Cells (HSCs)

HSC cells exist in the bone marrow and to a lesser extent in the peripheral blood and are responsible for the production of different blood lineages. They have been widely utilized in the treatment of hematological malignancies, but recently HSCs have been used in T1DM patients as well, mostly because of their immunomodulatory properties [31]. Several clinical trials used autologous HSC in T1DM patients and showed that HSC is safe and can improve glycated hemoglobin levels in a time‐dependent manner [39].

Stem cell therapy holds promise for T1DM by regenerating β‐cells and restoring immune tolerance. However, challenges such as differentiation efficiency, immune rejection, and teratogenicity remain, particularly in clinical applications. Recent trials, like VX‐880, have shown efficacy in patients with residual β‐cell function or recent‐onset disease, yet long‐term success in those with complete β‐cell loss is uncertain. Factors like immune response and disease duration influence outcomes, necessitating further research to optimize patient selection and enhance treatment efficacy across diverse T1DM populations [40].

### 3.4 Immunotherapy

Immunotherapy in T1DM aims to regulate abnormal immune responses responsible for β‐cell destruction. Unlike conventional treatments that only manage symptoms, immunotherapy seeks to alter disease progression by inducing immune tolerance, preserving β‐cell function, and potentially restoring pancreatic health [41].

#### 3.4.1 Epitope‐Based Precision Immunotherapy

Precision immunotherapy selectively targets autoreactive immune components while minimizing systemic immunosuppression. Traditional biologics focus on specific immune pathways but often lack specificity for autoimmune cells [42]. Antigen‐specific immunotherapies (ASITs) modulate immune tolerance by selectively engaging autoreactive cells [43]. However, challenges such as local inflammation and genetic variability complicate tolerance induction. ASITs are most effective when residual β‐cells are present; otherwise, patients require exogenous insulin therapy. Pancreatic islet transplantation offers an alternative, but limitations such as donor scarcity and strong immune responses hinder its success. A promising approach involves autologous stem cell‐derived β‐cells, yet these cells remain susceptible to autoreactive T cells [44].

#### 3.4.2 Suppress β‐Cell Autoimmunity

Combination therapies integrating immunomodulators with β‐cell regenerative agents are being explored to suppress β‐cell autoimmunity [45]. Targeting stem‐like autoimmune progenitors in pancreatic draining lymph nodes is a novel avenue for immunotherapy. Additionally, microneedle‐based intradermal administration of proinsulin peptides conjugated with gold nanoparticles enhances antigen‐specific tolerance [46].

Promising candidates for β‐cell protection include:

- Liraglutide and phosphatidylserine (PS): Demonstrated efficacy in reducing hyperglycemia in T1DM models [47].
- Epigenetic targets: MBD2 and RNLS influence Th1 program homeostasis and β‐cell resilience [48].
- Circulating CXCR5−PD‐1hi Tph cells: Serve as potential biomarkers and immunotherapy targets [49].
- Multi‐peptide immunotherapy: Administered intravenously, shows potential in restoring immune regulation in early‐stage T1DM, particularly in HLA‐DRB1*0401 carriers [50].

#### 3.4.3 Designing Personalized ASITs

Personalized immunotherapies leverage insights from cancer immunotherapy, β‐cell autoantigens, and HLA‐specific antigen identification [51]. Selecting HLA‐appropriate autoantigens could optimize regulatory responses while minimizing inflammatory side effects [52]. Emerging targets include:

- *Calcitonin gene‐related peptide (CGRP)*: A potential T1DM autoantigen with implications for diabetic neuropathy [53].
- *Sequential combination therapies*: Designed based on disease stage to enhance therapeutic synergy [53].

Immunotherapy therapy shows promise in rodent models, but translation to humans presents challenges due to immune system differences and vector‐related immunogenicity [54]. Human immune responses to viral vectors may diminish efficacy, necessitating safer delivery systems. Recent clinical trials, such as IMCY‐0098 (Phase 1b), highlight the potential of precision immunotherapy in early‐stage T1DM [55]. However, further research is required to optimize treatment strategies, particularly for patients with advanced disease stages [56].

### 3.5 Monoclonal Antibody (mAb) Therapy

mAbs have gained attention as a therapeutic strategy for T1DM by targeting immune cells involved in autoimmune‐mediated β‐cell destruction. These antibodies can deplete pathogenic immune effectors, reducing further tissue damage. Both T and B cell populations have been targeted in preclinical and clinical studies, demonstrating temporary benefits in disease management [57].

#### 3.5.1 B‐Cell Targeting mAbs

While T1DM is primarily a T cell‐mediated disease, B lymphocytes play a role by presenting antigens and activating T cells. Rituximab, an anti‐CD20 antibody used in hematological malignancies, has been investigated for T1DM. Studies have shown that Rituximab improves metabolic control, slows β‐cell loss, and reduces insulin requirements over 12 months [58]. However, patients do not achieve insulin independence, indicating that B‐cell depletion alone is insufficient to restore β‐cell function [59].

#### 3.5.2 T‐Cell Targeting mAbs

T‐cell targeting mAbs have emerged as a promising avenue in the treatment of T1DM. Abatacept is a cytotoxic T lymphocyte‐associated antigen 4 immunoglobulin fusion protein (CTLA‐4) that is intended to bind specifically to CD80/86 and prevent naive T lymphocytes from becoming activated early and proliferating. Compared to general immunosuppressants, abatacept is a more focused way to inhibit T cell activation because effector memory T cells are less reliant on CD28 co‐stimulation [60]. There are still ongoing recent clinical trials, such as NCT04118153 (involving abatacept) and NCT03929601 (evaluating abatacept in combination with rituximab), conducted in 2021 and 2022 investigating whether abatacept may have a more potent impact on the disease if administered at earlier stages of T1DM pathogenesis. Another T‐cell targeting mAb is Anti‐CD3 therapy, which targets the chain of the CD3 receptor on the T‐cell surface and is currently the most effective therapeutic target in altering the course of T1DM. Teplizumab is a humanized anti‐CD3 mAb that has been demonstrated to be the most effective treatment for T1DM progression slowing. Teplizumab has been shown in several human clinical trials to be an effective way to postpone the decline in C‐peptide production and can aid in maintaining β‐cell function [61, 62] and its effect can be sustained by an average of 15.9 months in T1DM [63]. Additionally, the Protegé research [64, 65], which enrolled 516 T1DM patients and treated them with teplizumab, showed that anti‐CD3 medication delayed the reduction of insulin secretion, induced disease regression, and resulted in 5% of the patients becoming insulin independent.

Additional studies to examine the impact of anti‐CD3 treatment in T1DM prevention were conducted as a result of these encouraging results. The effectiveness of teplizumab in delaying the onset of T1DM was recently shown by the findings of a Phase III follow‐up trial that included nondiabetic patients at high risk of developing T1DM, defined as having impaired glucose tolerance and at least two diabetes‐specific autoantibodies [66]. Even after a 7‐year follow‐up period, the C‐peptide levels of individuals who responded to treatment remained noticeably improved [67] Also, clinical responders to teplizumab therapy displayed a notable decrease in circulating CD4^+^ effector memory T cells as well as a reduction in the activation and regulatory gene expression in circulating CD8^+^ central memory T cells [68]. Teplizumab is now being studied in patients with recent‐onset T1DM (NCT03875729 and NCT04598893) and at‐risk persons (NCT04270942).

On November 17, 2022, the US Food and Drug Administration (FDA) approved teplizumab in stage 3 T1D patients. Initially, it showed that the drug could delay the stage 3 T1D onset by over 2 years in some patients [69]. Follow‐up results further supported the benefits of teplizumab in delaying T1D in high‐risk patients [70]. Additional clinical trials are ongoing and should provide further insight into whether the delay in T1D onset can be extended for a longer period. This therapeutic approach could offer new hope to patients with T1D and have a significant impact following early diagnosis.

mAb therapies for T1DM offer potential by specifically targeting immune cells involved in β‐cell destruction. Antibodies like Rituximab and Teplizumab improve metabolic control and delay disease progression, but long‐term results and safety in humans require further investigation.

### 3.6 Sodium‐Glucose Cotransporter‐2 (SGLT‐2) Inhibitors

SGLT2 inhibitors, designed initially for T2DM, have proven effective in managing glycemic control independently of insulin secretion [71]. Clinical studies in T1DM demonstrate reduced HbA1c, lower glucose variability, increased time in the optimal glucose range, and additional benefits such as weight reduction and decreased insulin dose [72].

The mechanism involves blocking the SGLT‐2 transporter in the renal tubule, inducing glycosuria and natriuresis. While these inhibitors show promise in both T1DM and T2DM, their use in T1DM is tempered by an increased risk of diabetic ketoacidosis (DKA), a serious adverse event. DKA risk varies across trials, leading to diverse regulatory stances, ranging from approval for T1DM with inadequate glycemic control to restrictions based on body mass index (BMI). Empagliflozin, a specific SGLT2 inhibitor, exhibits positive effects on HbA1c, body weight, and insulin use in T1DM, even at very low doses, with a lower DKA risk observed [73].

Dapagliflozin is the first SGLT2 inhibitor authorized for T1DM, recognized as a cost‐effective treatment option by health organizations [72]. Clinical trials, including DEPICT and EASE programs, consistently demonstrate improved HbA1c, reduced body weight, and insulin dose, despite an increased risk of genital infections. DKA risk mitigation strategies, including patient education, careful patient selection, and monitoring, are crucial for safe SGLT2 inhibitor use in T1DM [74].

SGLT2 inhibitors present a risk of urogenital infections, particularly mycotic genital infections, but show equipoise in urinary tract infections. Adverse events include the rare Fournier's gangrene, with a reported increase in cases compared to other diabetes drugs. DKA, a serious complication associated with SGLT2 inhibitors, necessitates thorough education for patients and healthcare professionals to recognize early signs of metabolic decompensation. Selection of appropriate candidates, based on factors such as BMI, insulin needs, and adherence, is crucial to mitigate DKA risk [72].

### 3.7 Dipeptidyl Peptidase‐4 (DPP‐4) Inhibitors

DPP‐4 inhibitors, initially designed for T2DM, are emerging as promising therapeutic agents for T1DM. These inhibitors elevate endogenous GLP‐1 levels, enhancing insulin secretion, suppressing glucagon production, and contributing to antihyperglycemic effects. Clinical trials and studies in T1DM animal models suggest their potential to alleviate insulitis, stimulate β‐cell proliferation, and delay autoimmune responses [75]. DPP‐4 inhibitors exhibit high efficacy, inhibiting DPP‐4 by > 80%–90%. Administered once daily or once weekly, they offer good bioavailability. Clinical studies establish their non‐inferiority compared to other antidiabetic agents, proving well‐tolerated, body weight neutral, and safe even in impaired kidney function [76]. Promising results in small‐scale trials indicate improved glycemic control, reduced insulin requirements, and attenuated β‐cell destruction. Larger, long‐term trials are needed. DPP‐4 inhibitors demonstrate safety in T1DM, with minimal side effects. Meta‐analyses indicate no significant alterations in hypoglycemia risk when combined with insulin [77].

Phase III studies highlight good safety and tolerability, with common adverse events rarely leading to treatment discontinuation. A low risk of acute pancreatitis is associated with DPP‐4 inhibitor therapy. Concerns about cancer risk, including pancreatic cancer, are not substantiated. Bullous pemphigoid, a rare skin disease, is associated with vildagliptin, but overall, the class maintains a favorable safety profile. In summary, DPP‐4 inhibitors exhibit potent immune modulation, promising therapeutic effects in T1DM, and a favorable safety profile, marking a significant contribution to the evolving landscape of diabetes management [76].

## 4 Biomarkers for Treatment Response

While insulin therapy remains the mainstay of T1DM treatment, there is a growing interest in identifying biomarkers that can predict treatment response and guide personalized therapeutic strategies. Although most of the studied biomarkers were useful in the prevention and early detection of T1DM, there are some metabolic and immunologic biomarkers that can be used to monitor treatment response in diabetic patients. The most common metabolic biomarkers are glycemic variability [78], advanced glycation end products (AGEs) [79], ketone bodies [80], and measuring β‐cell function [81]. Besides metabolic biomarkers, immunological biomarkers are measurable indicators of T1DM. Some of the useful biomarkers are as follows:

### 4.1 Autoantibodies

Autoantibodies targeting specific pancreatic islet antigens, such as insulin, GAD, and islet antigen‐2 (IA‐2), are hallmark features of T1DM. The presence and persistence of these autoantibodies can help identify individuals at risk of developing T1D, but in the case of therapeutic outcomes, there are controversies about the usefulness of autoantibodies. Some studies reported the predictive value of autoantibodies in response to immunotherapy [82, 83] while other studies did not find a correlation between the level of autoantibodies and response to treatment [84, 85].

### 4.2 T‐Cell Subsets

T cells play a crucial role in the autoimmune response against pancreatic β‐cells in T1DM. Several assays such as enzyme‐linked immunospot (ELISPOT), major histocompatibility complex (MHC) I or II tetramers, and immunoblot have been developed to study the frequency of autoantigen‐specific T cell subsets including CD4^+^, CD8^+^, and regulatory T cells in patients, particularly after biological therapies. Reproducibility and standardization are the main obstacles in their clinical use. On the other hand, different studies have shown conflicting results, possibly because peripheral blood does not reflect tissue‐resident cells [84].

### 4.3 Cytokines

Cytokines are signaling molecules that regulate immune responses. Cytokines are classically classified into anti‐and pro‐inflammatory groups, which can induce regulatory functions to prevent βcell damage or lead to T1DM onset and progression by promoting diabetogenic immune cells. Many cytokines have been studied in the case of T1DM, but as they have pleiotropy and their functions are context related, the result is contradictory. For example, two recent studies showed that higher levels of IL‐6 and IL‐21 are associated with a better response to monoclonal therapies [59] while another study reported no improvement after the blockade of the IL‐6 receptor [86]. In this regard, there is no valid, useful cytokine marker for evaluating the response to therapy in T1DM yet.

## 5 Future Directions

In our view, the treatment landscape for T1DM is advancing with promising therapies like mAbs, SGLT2 inhibitors, and DPP‐4 inhibitors. While these treatments improve glycemic control and preserve beta‐cell function, challenges remain in achieving long‐term effectiveness. mAbs such as teplizumab and abatacept show potential in immune modulation but require more long‐term studies. SGLT2 inhibitors offer metabolic benefits but carry a risk of DKA. DPP‐4 inhibitors show promise in preserving beta‐cell function but need further clinical trials. Moving forward, combination therapies, personalized treatment strategies, and a better understanding of immune modulation will be essential for advancing T1DM management.

## 6 Conclusion

In conclusion, the landscape of T1DM treatment is undergoing a profound transformation with the exploration of innovative therapies and novel approaches. From SGLT‐2 and DPP‐4 inhibitors to gene therapy, stem cell therapy, and immunotherapy, a diverse spectrum of interventions is emerging. These advancements bring promise for improved glycemic control, β‐cell regeneration, and immune modulation. Challenges persist, but ongoing research and collaborative efforts position T1DM care at the forefront of precision treatment. The evolving strategies offer hope for enhanced outcomes and a brighter future for individuals grappling with the complexities of this autoimmune condition.

## Conflicts of Interest

The authors declare no conflicts of interest.

## Data Availability

All readers are advised to communicate with the corresponding author for any additional inquiries.
